# The Emerging Applications of Artificial MicroRNA-Mediated Gene Silencing in Plant Biotechnology

**DOI:** 10.3390/ncrna11020019

**Published:** 2025-03-02

**Authors:** Luis Alberto Bravo-Vázquez, Ana Marta Castro-Pacheco, Rodrigo Pérez-Vargas, Joceline Fernanda Velázquez-Jiménez, Sujay Paul

**Affiliations:** School of Engineering and Sciences, Tecnologico de Monterrey, Queretaro 76130, Mexico

**Keywords:** artificial miRNAs, gene silencing, plant breeding, crop improvement, plant stress, functional genomics

## Abstract

Improving crop yield potential is crucial to meet the increasing demands of a rapidly expanding global population in an ever-changing and challenging environment. Therefore, different technological approaches have been proposed over the last decades to accelerate plant breeding. Among them, artificial microRNAs (amiRNAs) represent an innovative tool with remarkable potential to assist plant improvement. MicroRNAs (miRNAs) are a group of endogenous, small (20–24 nucleotides), non-coding RNA molecules that play a crucial role in gene regulation. They are associated with most biological processes of a plant, including reproduction, development, cell differentiation, biotic and abiotic stress responses, metabolism, and plant architecture. In this context, amiRNAs are synthetic molecules engineered to mimic the structure and function of endogenous miRNAs, allowing for the targeted silencing of specific nucleic acids. The current review explores the diverse applications of amiRNAs in plant biology and agriculture, such as the management of infectious agents and pests, the engineering of plant metabolism, and the enhancement of plant resilience to abiotic stress. Moreover, we address future perspectives on plant amiRNA-based gene silencing strategies, highlighting the need for further research to fully comprehend the potential of this technology and to translate its scope toward the widespread adoption of amiRNA-based strategies for plant breeding.

## 1. Introduction

In recent decades, there has been a significant trend toward enhancing plant production and developing improved plant species, as both strategies are critical for ensuring food security. This is particularly crucial due to the constant growth of the global population and decreases in agricultural land, the emergence of devastating plant diseases, and global warming [1,2,3]. Therefore, developing novel breeding methodologies is essential to reduce the time, resources, and space required for enhancing agricultural efficiency and sustainability [4,5,6]. In this context, the exploitation of RNA interference (RNAi) technologies has been identified as a potential approach to selectively inhibit the expression of target genes in important plant species with the aim of generating crops resistant to biotic and abiotic stresses, enhancing nutritional quality and improving various aspects of plant physiology and biochemistry (e.g., flowering time, plant structure, and the production of secondary metabolites) [7,8].

RNA silencing is a gene regulatory system that is evolutionarily conserved and naturally present in eukaryotic cells. This regulatory system of gene expression is orchestrated by endogenous small RNAs (sRNAs), which are divided into two predominant classes: endogenous small interfering RNAs (siRNAs) and microRNAs (miRNAs) [9,10]. miRNAs are short, non-coding RNA (ncRNA) molecules, typically 20–24 nucleotides (nt) in length, that regulate gene expression post-transcriptionally by binding to complementary sequences present in the target messenger RNA (mRNA) and lead to the repression of gene expression via mRNA cleavage or translation inhibition [11,12]. Therefore, the role of miRNAs as master regulators of gene expression possesses key implications in diverse biological phenomena, such as development [13,14,15], reproduction [16], disease resistance [17], stress response [18,19], cell differentiation [20], as well as secondary metabolism [21,22].

Mechanistically, during plant miRNA biogenesis, miRNA genes (*MIRs*) are transcribed by the RNA polymerase II (Pol II) enzyme into primary miRNA transcripts (pri-miRNAs) [23,24,25,26,27,28,29,30]. The pri-miRNAs are then processed in the nucleus by the microprocessor complex, which includes the RNA-binding protein HYPONASTIC LEAVES 1 (HYL1), Dicer-like 1 (DCL1), and SERRATE (SE), to generate precursor miRNAs (pre-miRNAs) [23,24,25,26,27,28,29]. Pre-miRNAs are processed into miRNA/miRNA* duplexes, and their stability is enhanced at the 3′ ends through methylation by HUA ENHANCER 1 (HEN1) [23,24,25,26,27,28,29,30]. Exportation of pre-miRNAs to the cytoplasm is usually attributed to HASTY (HST) [26,27,31,32,33]; nevertheless, it has been shown that HST is not essential for miRNA export in plants [34,35,36,37]. Indeed, the site of RNA-induced silencing complex (RISC) assembly remained unclear for several years until Bologna et al. [38] demonstrated that this complex is predominantly assembled within the nucleus and subsequently exported to the cytoplasm in a chromosomal maintenance 1 (CRM1)/nuclear-export signal (NES)-dependent manner. During RISC assembly, the guide strand of mature miRNAs is loaded into an ARGONAUTE (AGO) protein to target mRNAs for degradation or translational repression [23,24,25,26,27,28,29,30]. Concurrently, the other strand, known as the passenger strand or miRNA*, is usually degraded [23,27,29,39]. However, increasing evidence indicates that miRNA*s could also have important regulatory roles in plants [40,41,42,43,44,45].

miRNAs have been modified as part of the development of artificial miRNA (amiRNA) technology, which now forms a valuable tool for elucidating the functions of endogenous miRNAs and their target mRNAs (transcribed by the corresponding target genes); in fact, plant amiRNAs can be designed to target nucleic acids of interest with higher selectivity when compared to endogenous plant miRNAs [46,47]. Some of the pioneers in the field of plant amiRNA-based technology were Vaucheret et al. [48]. In their study, these researchers demonstrated that developmental defects caused by a miR168-resistant AGO1 mRNA could be rescued by an engineered amiRNA complementary to the mutant AGO1 transcript. This confirmed the regulatory role of miR168 and showcased the potential of amiRNAs as precise tools for gene silencing and exploring miRNA functions in plants [48]. Hence, since amiRNAs mimic the structure and functions of natural miRNAs, this allows researchers to study gene regulation pathways and mechanisms [49,50,51,52,53,54]. Additionally, amiRNAs exhibit practical applications in agriculture, where they can be used to improve plant resistance to various biotic and abiotic stresses, including drought, salinity, insect pests, viruses, fungi, and bacteria [55,56,57,58,59,60,61].

amiRNAs can be expressed as transgenes by employing conventional plant transformation methods, such as *Agrobacterium tumefaciens*-mediated transformation. This approach allows the integration of the amiRNA expression cassette into the plant genome, resulting in stable transgenic lines [46,62]. These lines have the potential to express the amiRNA across multiple generations, enabling the long-term and heritable gene silencing of a particular target gene [63,64]. This method is predominantly useful for studying gene function or developing plants with enhanced traits that require consistent amiRNA expression. Alternatively, amiRNAs can be delivered exogenously for transient expression [65]. In this case, amiRNAs are applied directly to plant cells or tissues, typically using techniques such as agroinfiltration [66,67] or nanoparticle-mediated delivery [68,69]. Unlike transgenic approaches, the exogenous delivery of amiRNAs does not integrate the amiRNA sequence into the plant genome, resulting in a temporary effect that lasts only for the duration of the amiRNA’s stability within the plant. This method is advantageous for rapid functional analyses or applications where stable transformation is not feasible [70].

The application of amiRNAs in plant biotechnology offers significant advantages over other technologies, such as clustered regularly interspaced short palindromic repeats (CRISPR)/CRISPR-associated enzyme (Cas) and transcription activator-like effector nucleases (TALENs). For instance, amiRNAs represent cost-effective and accessible tools for researchers [51,71,72,73]. In addition, amiRNAs can be designed to target multiple genes simultaneously, allowing for the modulation of complex traits [67,74,75,76,77]. The use of amiRNA-based gene silencing also offers enhanced precision and minimizes unintended off-target interactions [51,61,78,79]. High compatibility with RNA promoters, tailored precision, and the capacity to degrade target genes without impacting the expression of non-targeted genes are other examples of the significant advantages of amiRNAs [51,80]. Another noteworthy aspect is that, as mentioned above, amiRNAs can be transiently expressed without the need for integration into the plant genome [67,81,82], thereby aiding in overcoming controversies surrounding transgenic crops [83].

Consistently, the utilization of plant amiRNAs could hold significant value in unraveling the biological roles of endogenous miRNAs and genes in plants and enhancing the development of novel amiRNA-mediated protocols for the tailored breeding of important plants. Hence, in this review, we offer a broad overview of the recent experimental evidence concerning the amiRNA-based manipulation of gene expression in plants and discuss the future prospects of plant amiRNAs, emphasizing the necessity for additional research to completely exploit the potential of this technology and facilitate its widespread adoption in plant improvement programs.

## 2. The Essentials of amiRNA Design

**Figure 1:**
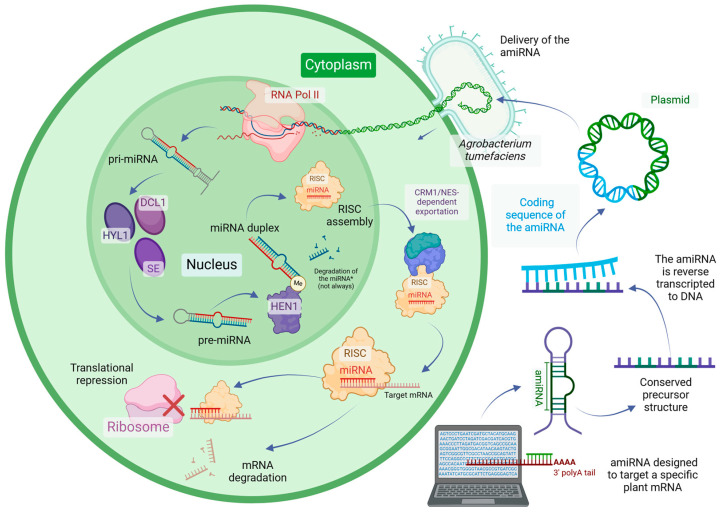
Overview of the design and biogenesis of amiRNAs. Initially, amiRNA candidates targeting a specific mRNA are generated using computational methods. These amiRNAs are then delivered to the plant in the form of precursors containing the sequence of the amiRNA. Afterward, the endogenous miRNA processing machinery of the plant cell (described in the Introduction Section) transforms the precursors into functional amiRNAs capable of silencing the expression of the gene or genes of interest. The term miRNA* refers to the passenger strand that is part of the miRNA duplex, whereas the term miRNA specifically denotes the guide strand, which typically associates with the RISC to execute its regulatory functions (created with a licensed version of Biorender.com).

The numerous benefits of using amiRNAs to silence genes have turned them into prominent tools in genetic engineering. Nonetheless, cases of failure and inefficiency in amiRNA-mediated gene silencing can occur, primarily arising from a limited comprehension of the mechanisms of miRNA processing. Therefore, it is crucial to broadly understand the mechanisms underlying miRNA biogenesis and miRNA-induced gene silencing when designing amiRNAs [48,52,62,84,85]. On this basis, amiRNAs are designed by modifying an endogenous miRNA precursor. In this process, the region of the precursor that contains the mature miRNA sequence is replaced with an amiRNA sequence complementary to the nucleic acid of interest, allowing the amiRNA to specifically target it. Preserving the secondary structure of the endogenous precursor guarantees that the plant’s miRNA biogenesis machinery will recognize and process the modified precursor, resulting in the production of a functional, mature amiRNA [50,85,86,87]. Figure 1 illustrates the general pipeline of amiRNA design and biogenesis.

For instance, the *Arabidopsis* miR319a and rice miR528 precursors serve as popular expression backbones for amiRNAs as they have been extensively utilized in creating loss-of-function mutants in both dicot and monocot plant species due to their widespread conservation throughout the plant kingdom [72,88,89,90,91,92,93,94]. As a matter of fact, it is crucial to keep the pre-miRNA backbone as intact as possible since the disruption of its stem-loop structure may produce detrimental effects on mature miRNA processing, thereby affecting the expression of the crafted amiRNA [50,85,94]. For an amiRNA candidate to be considered suitable, it must exhibit perfect or near-perfect base pairing with the sequence of the target gene, and it has been recommended that the amiRNA should have a uracil (U) residue located at position 1 from the 5′ end [95,96,97]. Nonetheless, compelling evidence indicates that AGO1 can effectively incorporate miRNA guide strands with 5′ terminal bases different from U, or alternatively, that other AGO proteins like AGO2, AGO4, and AGO5 can recruit mature miRNAs with other 5′ terminal nucleotides to mediate RNA silencing [98,99].

As previously stated, the effectiveness of plant miRNAs is primarily driven by the degree of complementarity with their target sequence rather than by the specific location of the target binding site on the mRNA. Notwithstanding this, plant miRNAs predominantly mediate mRNA cleavage by targeting coding regions or open reading frames (ORFs), in contrast to human miRNAs, which mostly bind to 3′ untranslated regions (UTRs) [100,101]. However, it has been demonstrated that some plant miRNAs can regulate mRNA expression by binding to either the 3′ or 5′UTRs [102,103,104], highlighting the flexibility of miRNA-mediated regulation of plant expression in plants. Other features that have been shown to be important in the production of plant miRNAs are the inclusion of cytosine (C) or guanine (G) at positions 8–9 and 18–19 and adenine (A) or U in positions 5, 7, 10, and 15. Additionally, less strict signatures include G/C at positions 2–4, 6, and 21 and A/U at positions 17 and 20 [105]. Remarkably, amiRNAs presenting these signatures were abundantly produced in transformed *N. tabacum* plants [105]. Hence, these sequence signatures could be considered during the design of plant amiRNAs.

On the other hand, a thorough off-target analysis should be conducted while designing amiRNAs by performing a BLAST search against the entire genome sequence of the target plant species [96,106,107]. It is worth mentioning that the biogenesis of phased small interfering RNAs (phasiRNAs) is usually, but not exclusively, mediated by miRNAs of 22 nt. In general, miRNAs trigger phasiRNA production by cleaving single-stranded target RNAs, such as mRNAs and long non-coding RNAs (lncRNAs), that are then converted into double-stranded RNAs (dsRNAs) by an RNA-dependent RNA polymerase [108,109,110,111,112]. Subsequently, such dsRNAs are processed by DCL enzymes into phasiRNAs. At the end, phasiRNAs are loaded onto AGO proteins to guide RNA silencing [108,109,110,111,112]. Since phasiRNAs can silence genes that were not originally considered in the amiRNA design, to prevent the unintended production of phasiRNAs from amiRNAs, it is advisable to design amiRNA sequences with 1–3 mismatches between the miRNA and its target mRNA [113].

Consistently, by comprehending the intricate mechanisms underlying miRNA production, researchers can design amiRNAs that mimic endogenous ones, thereby regulating target gene expression with precision. This knowledge is especially valuable for non-model plants, where genetic manipulation techniques are less established compared to model species. Notably, a specialized web application is currently available for designing amiRNAs, which is known as Web MicroRNA Designer (WMD) [49]. This platform has been widely used in several studies included in this review to successfully create plant amiRNAs, validating the effectiveness of this tool.

## 3. Advances in the Development and Improvement of Plant amiRNAs

amiRNAs have witnessed significant advancements, becoming promising tools for researchers focused on gene silencing and functional genomics across a wide range of plant species. Significantly, methods for designing amiRNAs have significantly evolved over the years. In 2014, an innovative approach for the creation of amiRNAs from the *A*. *thaliana MIR390a* backbone was defined and tested by Carbonell et al. [73]. Some of the key reasons to choose *AtMIR390a* as a precursor backbone for amiRNA design comprise the accuracy with which it is processed, its high conservation across plant species, and its foldback with a relatively short distal stem-loop. The strategy proposed involves the insertion of amiRNA sequences via annealing two overlapping, partially complementary oligonucleotides, which are then directionally ligated into a *Bsa*I/*ccdB*-based vector designed for zero background cloning. These *Bsa*I/*ccdB* vectors, optimized for amiRNA expression, incorporate modified *Arabidopsis MIR390a* precursors where part of the native sequence has been replaced by a *ccdB* cassette bordered by *Bsa*I sites, facilitating efficient and precise amiRNA cloning. This methodology was validated in a transient expression assay performed in *N*. *benthamiana*, where six crafted amiRNAs originated from *AtMIR390a* precursor foldbacks were principally accumulated as 21 nt species, indicating high levels of accurately processed amiRNAs. Moreover, the functionality of this approach was confirmed in *Arabidopsis* using the amiRNAs amiR-Ft, amiR-Lfy, and amiR-Ch42, which reduced the expression levels of their corresponding mRNA targets [73].

A study carried out by Guo et al. [114] highlighted the importance of validating amiRNAs that do not generate unintended sRNAs, as these can cause off-target effects. These authors designed the amiRNA amiRchs1 (based on *Arabidopsis* pre-miR319a) to silence *CHS* genes in *Petunia hybrida* (petunia) and used a modified 5′ RACE technique to track cleavage sites and processing intermediates, confirming that CHS mRNAs were cleaved as intended and that the precursor processed from loop to base. However, deep-sequencing analysis revealed that, besides the target amiRNAs, additional sRNAs from other regions of the amiRNA precursor accumulated with high frequency. These unintended sRNAs, which could potentially silence genes in petunia and other Solanaceae species, underscore the importance of rigorous validation to avoid unintended silencing. Importantly, the study found no secondary sRNA production from the designed amiRNA, even with perfect pairing to its target. This result reinforces the high specificity and effectiveness of perfectly complementary amiRNAs in plant gene silencing, highlighting their utility while stressing the need for careful off-target assessment in future applications.

In another investigation [115], a one-step procedure to construct amiRNA precursors was proposed to facilitate the use of amiRNA technology in *Chlamydomonas reinhardtii* for the screening of knockdown specimens. To create the amiRNA construct, a set of eight oligonucleotides, each containing 40–45 nt, was synthesized. Four served as the backbone (based on the precursor of cre-miR1157), while the other four were specific to the target gene. Since these oligonucleotides had overlapping regions, it enabled the formation of a double-stranded amiRNA precursor through a single annealing and ligation step, thus bypassing the traditional PCR amplification step used in classic amiRNA constructs in *Chlamydomonas*. The assembled oligonucleotides were then inserted into an expression vector. To monitor amiRNA expression, a luciferase gene (*G-Luc*) was positioned upstream of the amiRNA precursor. This design allows the transcript’s initial part to translate into luciferase while the rest is processed into the amiRNA, which targets the gene of interest. Luciferase activity thus serves as an indirect measure of amiRNA expression. Accordingly, these vectors could simplify the construction of amiRNAs and offer valuable tools for functional genomics in *Chlamydomonas*, as well as in other model organisms. In a similar approach, Zhang et al. [76] produced amiRNAs within a portable intron containing the GFP as a reporter gene. This setup allows the simultaneous production of both the fluorescent reporter and the amiRNA from the same transcript, enabling the reporter to act as a visible indicator of amiRNA activity in plants. In this report, transgenic plants selected based on fluorescence consistently exhibited optimal target gene silencing. Additionally, these researchers generated a strategy for multiplex gene silencing using endogenous tRNA-processing machinery.

The main challenge in amiRNA application is choosing effective amiRNAs from hundreds of bioinformatically generated candidates in the WMD to enable optimal target gene silencing at the protein level in vivo. On this basis, in 2014, a straightforward and widely adaptable method was developed by Li et al. [116]. This strategy was named the epitope-tagged protein-based amiRNA (ETPamir) screening method, and it involves expressing epitope-tagged proteins from one or more potential target genes alongside specific amiRNA candidates either continuously or under inducible conditions in plant mesophyll protoplasts, in which DNA transfection is highly efficient due to their lack of cell wall. By measuring the accumulation of tagged proteins using immunoblotting, researchers can assess the effectiveness of each amiRNA in silencing its target gene since they are correlated. This approach bypasses the challenges posed by limited plant antibodies and the intricate nature of plant amiRNA-mediated silencing at target mRNA and/or protein levels. In this way, constitutive expression of optimal amiRNAs can achieve a knockout effect on target genes, thereby inducing silencing phenotypes akin to genetic null mutants.

Techniques to produce a large number of amiRNA constructs quickly and affordably have been previously discussed with the cloning and synthesis of the amiRNA based on *AtMIR390a*-B/c expression vectors [73]. However, this was effective for eudicots. For this reason, in 2015, a similar method for monocot amiRNA constructs was optimized. Four amiRNAs derived from hybrid OsMIR390-AtL precursors, consisting of a basal stem from *OsMIR390* and a distal stem-loop from *AtMIR390a*, were validated in transgenic *Brachypodium distachyon* plants [117]. The chimeric precursor allowed higher accumulation levels and more accurate processing of amiRNA. A similar strategy to the one previously mentioned [73] was used to create OsMIR390-B/c-based vectors that allowed the direct cloning of amiRNAs. To evaluate the performance of amiRNAs produced from the hybrid precursor, amiR-BdBri1, amiR-BdCad1, amiR-BdCao, and amiRBdSpl11 were designed to silence specific target gene transcripts in *B. distachyon*. As a result, all the transgenic specimens showed elevated levels of amiRNAs and reduced levels of the targeted transcripts. Lastly, 5′ RLM-RACE analysis confirmed that amiRNAs were highly specific. Therefore, amiRNAs produced from chimeric OsMIR390-AtL precursors are likely to be quite specific and effective at silencing genes in monocot species [117].

As discussed earlier, several successful techniques using precursors from species like *A. thaliana*, *Oryza sativa*, and *C*. *reinhardtii* have been proposed. Later, Castro et al. [118] reported a methodology for designing amiRNAs from the *Vitis vinifera* miR319e backbone, as it is the most basic miRNA of the miR319 family in terms of size and structure. In this context, the amiR319e-GFP was designed to target the exogenous reporter gene GFP in transgenic *N. benthamiana*. This approach involves a two-stage PCR process utilizing long primers that overlap, facilitating the full production of pre-amiR319e-GFP suitable for recombination into Gateway vectors without any additional requirements. A strong silencing effect on the GFP phenotype was observed in the transgenic group carrying GFP + amiR319e-GFP compared to the group transformed with only GFP, where GFP fluorescence was visible. Characterization of the transformed plants was conducted through a modified 5′ RACE of GFP mRNA, revealing the presence of a partially truncated GFP mRNA molecule generated in planta at the 3′ end. Moreover, extensive sRNA sequencing confirmed the presence of the expected 21 nt miR319e-GFP species, along with other 22 and 24 nt species showing sequence homology with the anticipated amiRNA. The implications of these molecules were unknown; however, their presence suggests that the technique can trigger the generation of secondary siRNAs [118]. Overall, these results show a promising tool for gene function evaluation using amiRNA interference by utilizing the vvi-miR319e precursor as a backbone.

An effective amiRNA backbone based on a miRNA precursor of *Fragaria vesca* (wild strawberry) was developed in 2019 by Li et al. [119]. A basic amiRNA vector was created using the *F. vesca* miR166 precursor, chosen for its short length, simple secondary structure, and high expression levels. Therefore, amiRNAs based on this precursor were created, where amiR-GUS, amiR-GFP, amiR-NE, and amiR390 specific sequences substituted the mature miR166 sequence. In all the transgenic plant lines (strawberry and tobacco), the amiRNAs effectively reduced the expression levels of their targets, demonstrating that this strawberry-derived backbone is able to generate functional amiRNAs.

Functional structures of amiRNAs are typically created by replacing the miRNA and miRNA* of the precursor with the amiRNA/amiRNA* sequences through site-directed mutagenesis using four overlapping PCRs in two rounds. To simplify and accelerate the construction procedure of amiRNAs, modified *Arabidopsis* miR319a and rice miR528 precursors were engineered by introducing specific mutations within their single-strand RNA region. For pre-miR319a, the GAATTG and TCTTGA sequences were replaced with *EcoRI* and *XbaI* restriction sites, respectively, while for pre-miR528, the AGGTCT and GAAGTT sequences were substituted with *StuI* and *EcoRI* restriction sites, accordingly. These approaches facilitated one-step cloning of a single PCR product of the amiRNA/amiRNA* sequences after digestion and ligation. Subsequent functional analyses in protoplasts and transgenic plants evidenced that the amiRNAs generated with this methodology can be effectively processed and regulate the expression of their targets [94].

As observed, in the past decade, amiRNA backbones based on endogenous miRNAs from various species, such as *Arabidopsis*, rice, *Chlamydomonas*, and strawberry, have been developed. Similarly, strategies like EPTamir have streamlined the identification of functional amiRNAs, facilitating the use of amiRNA technology across diverse plant groups. Nevertheless, to extend the reach of this technology beyond commonly studied plants, it remains essential to continue developing effective backbones for less-studied plant species. In this matter, the use of artificial intelligence and machine learning [120,121] could play a valuable role in the next few years by aiding the design of novel amiRNA precursors.

## 4. Applications of amiRNAs Against Infectious Diseases

Plant infectious diseases are mostly caused by pathogens such as fungi, bacteria, and viruses, leading to plant damage and subsequently compromising food security and crop yield. There are currently various approaches against plant infectious diseases that can be physical, chemical, or biological, but they are costly and can harm people’s health as well as the environment. In this context, amiRNAs offer a promising alternative for enhancing plant resistance against infectious diseases by preventing the expression of vital target genes belonging to the infectious agent. Through this approach, environmental biosafety is not compromised. Other advantages of amiRNA-mediated strategies include their high specificity and facility for amiRNA design, and that they might be tailored against multiple pathogens at the same time, thus positioning them as a highly promising solution against infectious diseases.

Park et al. [122] studied *A*. *thaliana* miR400 to understand its role in response mechanisms against two pathogens, the bacterium *Pseudomonas syringae* pv. *tomato* DC3000 and the fungus *Botrytis cinerea*. Accordingly, they developed transgenic plants that overexpressed miR400 via the CaMV35S promoter (35S:MIR400). It was revealed that miR400 plays an important role in *Arabidopsis* plants’ defense response against *P. syringae* and *B. cinerea* by cleaving mRNAs of target genes that encode pentatricopeptide repeat (PPR) proteins since the expression levels of the At1g06580 (*PPR1*) and At1g62720 (*PPR2*) genes were considerably downregulated in miR400-overexpressing transgenic plants compared to wild type (WT) planta. PPR proteins play important roles in controlling RNA metabolism in the post-transcriptional regulation of gene expression, circadian clock, seed development, defense against pathogens, and heat stress response. After the transgenic plants were infected with the aforementioned pathogens, they presented noticeably acute disease symptoms in comparison to WT plants, indicating a higher susceptibility to both pathogens. In this study, *PPR2* knockdown mutants were also developed using an amiRNA-mediated knockdown strategy to study their response upon infection with *P. syringae* and *B. cinerea*. The results demonstrated that the *PPR2* mutants were more susceptible to the pathogens compared to WT plants. Additionally, the authors exhibited that the overexpression of miR400 and downregulation of *PPR2* caused plants to have a higher sensitivity to heat stress [122]. This study has proven the increased susceptibility of *Arabidopsis* against *P. syringae* and *B. cinerea* with the overexpression of miR400 and *PPR2* knockdown. Even so, more research is needed to fully understand how miRNA regulation of PPR proteins impacts plant responses against different pathogens.

Wheat dwarf virus (WDV) infection in wheat and barley is the cause of dwarf disease, which generates huge economic losses in crop production. WDV infection is carried out by leafhoppers (*Psammotettix alienus*) mostly during autumn and spring, meaning that any method implemented to increase the resistance of plants against WDV must be effective at low temperatures, around 12–15 °C. In this context, barley plants were transformed by Kis et al. [123] with a polycistronic amiRNA, generated using a mutagenized barley precursor, *hvu-MIR171MOD*, which has a stable secondary structure that can foster the generation of amiRNAs. Among ten secondary amiRNA candidate precursor structures (eight targeting the replicase and two targeting the viral movement protein-coding regions of the virus), the three best-performing ones, targeting the replicase viral RNA, were selected to create a polycistronic amiRNA precursor construct called VirusBuster171. Barley plants were transformed with the VirusBuster171 construct via *Agrobacterium*, subsequently infected with WDV, and kept at 12–15 °C. At 42 DPI, the control plants displayed dwarfing signs and viral RNA accumulation, detected through PCR and Northern blot analyses. Although the presence of the virus was identified in the line displaying low amiRNA expression, virus accumulation was not detected in any of the transgenic lines displaying moderate and high amiRNA expression. Successively, the control plants exhibited ongoing progression of common and severe WDV symptoms (such as chlorosis and stunting), as well as increased virus concentration. The transgenic line with moderate amiRNA expression showed WDV symptoms after 112 DPI. The presence of the virus was confirmed with PCR analyses but could not be detected by Northern blot analysis. The line that expressed VirusBuster171 at a higher level was completely resistant to WDV. Additionally, it was determined that the introduced trait could be consistently inherited over successive generations [123]. In sum, this amiRNA approach against WDV in barley plants resulted in effective viral resistance at low temperatures. In addition, as the level of expression of amiRNAs increased, so did the plants’ resistance level.

*Phytophthora infestans* is an oomycete pathogen responsible for *Solanum lycopersicum* (tomato) late blight disease. Luan et al. [124] studied the role of miR1918 in the interaction between tomato and *P. infestans*. Previously, it has been indicated that the sequence of miR1918 in *P. infestans* (pi-miR1918) is similar to the one present in tomato (sly-miR1918). After infection with *P. infestans*, the expression of sly-miR1918 was downregulated except at 72 h post-inoculation (HPI), and its target, the really interesting new gene (*RING*) finger gene, was upregulated in the first 24 HPI. In contrast, pi-miR1918 expression decreased slightly after 3 HPI in the pathogen. Ath-pre-miR159a, from *A*. *thaliana*, was used as a backbone to synthesize the amiRNA pri-amiR1918 and to study its effect in tomato plants. Tomato plants were transformed via *A*. *tumefaciens*. These plants showed upregulation of sly-miR1918 expression for the first 7 days and downregulation of miR1918 target genes. Three days later, their leaves and those of WT plants were infected with *P. infestans*. It was found that the leaves that overexpressed the artificial pi-miR1918 showed more severe disease symptoms (a greater number of necrotic cells, lesion sizes, and sporangia) than those of the WT plants. These outcomes suggested that amiR1918 overexpression amplified tomato plants’ susceptibility to *P. infestans* infection. Additionally, it was determined that miR1918 is involved in silencing target genes, specifically the *RING* finger gene, which plays a substantial role in ubiquitination involved in plant defense. This study demonstrated *P. infestans* can employ miRNAs to inhibit the expression of genes in their host organisms, in this case, tomato plants, reducing their resistance to infection by this pathogen [124].

Tomato spotted wilt virus (TSWV) affects various crops and is a highly significant viral pathogen because of its economic impact. Mitter et al. [125] created amiRNAs against TSWV targeting the nucleoprotein (N) and silencing suppressor (NSs) genes. A shortened *Arabidopsis* miR159a (athmiR159a) backbone (without the stem base extending beyond the mature miRNA), composed of 173 nt, was employed to express the amiRNAs. Additionally, they introduced wobble sequences at nucleotide positions 12 and 13 of the complementary strand of each amiRNA construct, imitating a mismatch in the miR159a sequence arrangement to study the effect of such a mismatch. The amiRNA constructs were expressed in *N. benthamiana* and *N. tabacum* (tobacco) plants through *Agrobacterium* infiltration. It was observed that the plants that expressed amiRNAs targeting the N gene of the virus were resistant to TSWV, remaining without symptoms. However, the plants that expressed amiRNAs targeting the NSs gene showed symptoms upon TSWV infection and accumulated high levels of the virus, indicating that these constructs failed to protect the plants. Additionally, it was determined that the mismatch at nucleotide position 12–13 does not affect amiRNA expression or resistance level against TSWV.

Sun et al. [126] designed three dimeric amiRNA precursor constructs (each had two pre-miRNAs) to confer resistance to rice (*O. sativa*) plants against rice stripe virus (RSV) and rice black-streaked dwarf virus (RBSDV). Each construct targeted different sections of the coat protein (*CP*) gene. Moreover, the endogenous rice precursor of Osa-MIR528 was utilized to generate the dimeric pre-amiRNAs: pamiR-M (containing pre-amiR-RSVM and pre-amiR-RBSDVM), pamiR-3 (containing pre-amiR-RSV3 and pre-amiR-RBSDV3), and pamiR-U (containing pre-amiR-RSVU and pre-amiR-RBSDVU), which targeted the middle segment, 3′ end, and 3′UTR region of the RSV and RBSDV *CP* gene, respectively. Applying *A. tumefaciens*-mediated transformation procedure, rice plants were transformed with the three expression vectors that contained the modified dimeric amiRNA precursors used to produce mature amiRNAs, targeting both viruses simultaneously. The outcomes revealed a direct relationship between the resistance levels observed in the transgenic plants and the expression levels of amiRNAs. This correlation was attributed to the downregulation of target viral RNAs mediated by the amiRNAs and their degradation with both amiRNAs and siRNAs. Furthermore, it was found that rice plants containing pamiR-U, which targeted the 3′UTR of the *CP* gene, exhibited the highest level of virus resistance. Finally, it was determined that transgene and amiRNA-mediated virus resistance could be reliably passed on to subsequent generations [126].

Ugandan cassava brown streak virus (UCBSV) and Cassava brown streak virus (CBSV) are responsible for Cassava brown streak disease (CBSD), which has a significant effect on cassava plant production. An amiRNA approach to control this disease was developed by Wagaba et al. [127], and it was evaluated in *N. benthamiana*. In this investigation, the amiRNAs were tailored to target the *P1*, *P3*, *CI*, *NIb*, and *CP* genes of CBSV and UCBSV, as well as the 3′UTR of both viruses. In transient expression assays carried out in leaf tissues, only the constructs amiR159a-P1[CBSV], amiR159a-P1[UCBSV], amiR159a-NIb[CBSV], and amiR159a-CP[UCBSV] produced detectable and significant amiRNA levels. Therefore, those constructs were used to produce stable transgenic *N. benthamiana* plants transformed via *A. tumefaciens*. The results showed that the transgenic plants with the amiRNA constructs targeting *P1* and *NIb* of CBSV had 25–65% resistance against CBSV and showed similar resistance levels against UCBSV. The transgenic plants for amiRNA constructs targeting *P1* and *CP* of UCBSV had more than 50% resistance against UCBSV but provided less effective resistance against CBSV; only one of the transgenic lines displayed more than 50% resistance against it. Targeting the sequences of *P1*[CBSV] and *NIb*[CBSV] genes proved to be more effective in combating both CBSV and UCBSV when compared to the ones of *CP*[UCBSV] and *P1*[UCBSV] genes. Overall, high resistance levels against CBSD were consistent with elevated amiRNA accumulation in the transgenic plants [127].

Carbonell and Daròs [128] studied the use of amiRNAs and synthetic *trans*-acting small interfering RNAs (syn-tasiRNAs) to interfere with infections by viroids. Viroids have small, non-coding, circular, single-stranded RNAs with a compact secondary structure and affect multiple plant species. amiRNA constructs were developed and tested with the *Potato spindle tuber viroid* (PSTVd)–*N. benthamiana* pathosystem. Six amiRNAs targeting PSTVd(+) and six PSTVd(−) RNAs were selected for the constructs and *N. benthamiana* plants were agroinfiltrated with them. The observations indicated that PSTVd had lower accumulation levels in transgenic plants containing PSTVD(+) and PSTVd(−) amiRNAs than in control samples. However, it was determined that amiRNAs targeting PSTVD(+) RNAs are overall more active than those targeting PSTVD(−) RNAs. This study revealed that the downregulation of PSTVd RNAs can be carried out by amiRNAs, making them a promising strategy to induce viroid resistance and control viroid infections in plants.

Two of the most significant viruses that affect orchids are Cymbidium mosaic virus (CymMV) and Odontoglossum ringspot virus (ORSV). Petchthai et al. [129] developed an amiRNA-centered approach against these pathogens, testing their constructs on *N. benthamiana* plants, which can also be infected by CymMV and ORSV. Using the *O. sativa* miR528 as a backbone, CymMV and ORSV amiRNAs targeting the gene region responsible for encoding the RNA-dependent RNA polymerase (RdRp) of either CymMV or ORSV were produced and placed in a single cassette named amiRNA-CymMV-ORSV. The amiR-CymMV expression levels in transgenic *N. benthamiana* plants led to the hindering of CymMV infection, preventing CymMV RNA accumulation, and providing a high resistance level upon infection with this virus. However, amiR-ORSV expression levels were undetectable and consequently, transgenic *N. benthamiana* plants displayed disease symptoms and were weakly resistant to ORSV infection. Against mixed infection, transgenic plants presented only ORSV symptoms, though not as severe as the ones on WT plants, supporting the previous results that indicated a high percentage of resistance to CymMV.

Another application of amiRNAs in combating plant infectious diseases was explored by the research conducted by Carbonell et al. [66]. This study investigated the ability of amiRNAs and syn-tasiRNAs to confer resistance against TSWV in *N. benthamiana*. First, the antiviral activity of amiRNAs was analyzed by targeting specific segments of the viral genome. These segments were Segment L (large), which codes for the RNA-dependent RNA polymerase; Segment M (medium), which codes for the putative movement protein NSm and the structural proteins Gn/Gc; and Segment S (small), which codes for the nucleocapsid N protein and the silencing suppressor NSs. For each Segment, five amiRNAs were tested, and it has been determined that the most effective amiRNAs were amiR-TSWV-L-3, amiR-TSWV-L-5, amiR-TSWV-M-1, and amiR-TSWV-M-3. Among these effective amiRNAs, the most reliable was amiR-TSWV-L-5, preventing the appearance of symptoms entirely, with the complete absence of TSWV particles validated via ELISA assays. Notably, amiRNAs targeting sites in Segment S were found to be ineffective [66].

Following this, a construct named 35S:syn-tasiR-TSWV was developed. This construct incorporated four syn-tasiRNAs derived from the most effective amiRNAs. These syn-tasiRNAs were integrated into the vector pMDC32B-AtTAS1c-B/c. A similar construct was made as a control using four syn-tasiRNA sequences corresponding to amiR-GUS-1 and amiR-GUS-2 introduced into the pMDC32B-AtTAS1c-B/c vector. To trigger syn-tasiRNA biogenesis, miR173a had to be expressed by 35:MIR173a, which was co-agroinfiltrated independently. Lastly, the antiviral effectivities of 35S:amiR-TSWV-L-5, which was the most effective amiRNA construct against TSWV, and syn-35S:syn-tasiR-TSWV were compared against two different TSWV isolates: PVR and LL-N.05. The results showed that most of the plants inoculated either with 35S:amiR-TSWV-L-5 or syn-35S:syn-tasiR-TSWV remained symptomless, although TSWV presence was detected by ELISA when infected with PVR and LL-N.05. These findings suggest that both amiRNAs and syn-tasiRNAs are equally capable of providing resistance against TSWV to *N. benthamiana* [66].

In another report, Carbonell et al. [130] applied the amiRNA-based technology in *S*. *lycopersicum* to generate resistance against TSWV. In this investigation, a plasmid containing four syn-tasiRNAs was tested to provide resistance against TSWV. More precisely, syn-tasiRNA-TSWV-1 and syn-tasiRNA-TSWV-2 were employed to target different sites of the TSWV-L (large) RNA segment, while syn-tasiRNA-TSWV-3 and syn-tasiRNA-TSWV-4 were utilized to target different sites of TSWV-M (medium) RNA segment. It is worth noting that the co-expression of a construct containing miR173 (35S:miR173) was required to trigger syn-tasiRNA biogenesis. Given that miR173 is absent in *S. lycopersicum*, the additional miRNA sequence was added downstream of the syn-tasiRNA-TSWV plasmid. At first, the 35S:syn-tasiRNA-TSWV/miRNA173 plasmid was agroinfiltrated into the model plant *N. benthamiana*. The results of this initial assay confirmed that 35S:syn-tasiRNA-TSWV/miR173 had effective antiviral activity as leaves agroinfiltrated with it had no signs of visible symptoms nor accumulation of TSMV. Next, the 35S:syn-tasiRNA-TSWV/miRNA173 plasmid was introduced and expressed in *S. lycopersicum*, the TSWV’s natural host. The accumulation of *syn-tasiRNA-TSWV* showed variability among different tomato lines. Indeed, the two lines with the lowest accumulation were the only ones that showed infection symptoms at 20 DPI. Further tests were conducted to compare syn-tasiRNA-TSWV-mediated multi-targeting of viral RNA resistance against the 35S:amiR-TSWV. This amiRNA expresses anti-TSWV amiRNA with the same sequence as syn-tasiR-TSWV-1. The outcomes indicated that amiRNA-TSWV was less effective than syn-tasiRNA-TSWV against TSWV. Intriguingly, syn-tasiRNA-TSWV demonstrated a lower concentration than amiRNA-TSWV, but syn-tasiRNA-TSWV displayed higher effectiveness, which could have been generated by the combined action of the four syn-tasiRNAs [130]. TSWV has a substantial economic impact on crops of global importance, like tomato and pepper, resulting in substantial losses worldwide. Therefore, this study is crucial as it offers in-depth research on a necessary enhancement of the amiRNA technology to confer resistance in tomato plants against TSWV.

In an additional investigation, Liang et al. [131] attempted to provide *N. bethamiana* with resistance against the cucumber green mottle mosaic virus (CGMMV). On this basis, they designed vectors containing amiRNAs that targeted the specific sequences of CP, movement protein (MP), and Rep proteins. The amiRNAs were named amiR1-CP, amiR2-CP, amiR3-MP, amiR4-MP, amiR5-Rep, and amiR6-Rep accordingly, reflecting the target of each amiRNA on its name. The vectors carrying these amiRNAs were later agroinfiltrated into *N. bethamiana*. The outcomes implied that the amiRNA that targeted the CGMMV CP was the most efficient in providing viral resistance against CGMMV, leading to a decreased viral accumulation. On the other hand, the amiRNA that targeted the CGMMV MP gene was the least efficient. CGMMV remains important since it is globally distributed and seed disinfection is inefficient in eliminating this virus. Furthermore, it has not received as much attention as other viral infections. Therefore, the prominence of developing an amiRNA-centered strategy to overcome the losses caused by CGMMV should not be overlooked.

Sharma and Prasad [132] designed an amiRNA that targeted the *AC1* gene of the tomato leaf curl New Delhi virus (ToLCNDV), a gene involved in the virus replication process. The amiRNA was delivered to tomato plants via *A. tumefaciens* and the AC1-amiRNA was successfully expressed. At 7 DPI, the modified tomato plants presented fewer symptoms contrary to non-transformed controls, which presented leaf curling. The modified plants displayed a reduced accumulation of viral DNA at 21 DPI when compared to untreated controls. The resistance of the modified plants ranged from 40 to 80%, which is higher than the natural known tolerance to ToLCNDV. Finally, the results obtained by the Northern blot technique showed that the overexpression of AC1-amiRNA led to the inhibition of virus accumulation through the reduction in expression levels of genes like AC3, AC2, and mainly AC1. Sanger sequencing demonstrated that no mutations were caused in the target site AC1 in AC1-amiRNA-modified plants. This factor is important, as these mutations would change the sequence of the target site, making it unrecognizable for the amiRNA. Also, fruits from the modified plants showed no variation in size or quantity, which was not the case with non-modified infected plants that showed fewer and smaller fruits as a result of the virus infection. This innovative amiRNA-based treatment is important as it explores the application of amiRNA technology to provide environmentally friendly protection against ToLCNDV to tomato plants.

Hu et al. [133] investigated the association between *P*. *infestans* and potatoes, as such a virus causes severe damage to this plant species. It was discovered that the main miRNA involved in the infection process is miR8788, whose target is an alpha/beta hydrolase-type encoding gene in potato (*StABH1*). Three different tests were then performed to inspect the relationship between *P. infestans* miR8788 and its target gene *StABH1*. Initially, *StABH1* was studied in transgenic potato lines modified to overexpress *StABH1*. This was performed to determine the disease response to *P. infestans*. At five DPI, results showed reduced disease lesions and less growth of *P. infestans* in inoculated leaves when compared to WT specimens. At that time, the *StABH1* levels were 10 times lower than uninfected plant lines. Afterward, different transgenic potato lines were generated containing an amiRNA, which induced *StABH1* slicing (*StamiRNA*). After inoculation with *P. infestans*, the virus spread quickly, forming sporangiophores at 2 DPI, which covered the leaves along with mycelia and sporangia at 3 DPI. *StamiRNA* potato lines presented a higher concentration of *P. infestans* DNA and lower expression levels of the *StABH1* transcript level when compared to the WT strains. The results obtained by this work provide a novel insight into the importance of miR8788 in *P. infestans* infection.

In an attempt to generate resistance against zucchini yellow mosaic virus (ZYMV), Berbati et al. [134] designed four different amiRNAs derived from the gene *HC-Pro*, which encodes a protein involved in aphid transmission of the virus. These amiRNAs targeted different regions within the *HC-Pro* gene: positions 115–135 and 1162–1182 were targeted by amiZYMV_HC-115s and amiZYMV_HC-1162s, respectively (the “s” in the names indicates sense). The other two amiRNAs, amiZYMV_HC-182as, and amiZYMV_HC-196as, targeted positions 162–182 and 176–196, respectively (with “as” denoting antisense orientation). The amiRNAs were then inserted into the vector AtMIR390aB/c, and their effects were subsequently evaluated in zucchini and luffa plants, as well as in *N. benthamiana*. The *N. benthamiana* plants used in this study had a DCL4 knockdown mutation that allowed ZYMV infection despite the fact that *N. benthamiana* is a non-natural host. The results showed that the amiRNA with the highest effectiveness was amiZYMV_HC-196as since it induced a 31% protection value in luffa and a 42.5% protection value in zucchini against ZYMV. The enhanced efficacy of amiZYMV_HC-196as can be attributed to its proximity to a structural bulge in the target sequence. This study underscores the potential application of amiRNA technology against ZYMV infection, supporting sustainable and environmentally safe agricultural practices.

The application of amiRNAs to combat plant pathogens has widely been explored, yielding promising results for this technology. Undoubtedly, this technology could not only enhance crop yield and food production but also mitigate economic losses associated with plant diseases. However, one of the main challenges ahead lies in scaling this approach to match the vast hectares of crops planted annually. Therefore, significant efforts must focus on designing strategies that enable the creation of pathogen-resistant plants via amiRNA-based technology on the large scale required for agricultural fields.

## 5. Applications of amiRNAs Against Insect Pests

Current agricultural pest management practices are often associated with negative impacts, including environmental pollution, pesticide resistance, and harmful effects on beneficial species, humans and pests alike. However, plants need to defend themselves against insect pests while attracting pollinators and beneficial insects. Biotechnological approaches, particularly those centered on RNAi, show promise in pest management by exploiting the role of amiRNAs in plant–insect interactions. Indeed, amiRNAs can act at the post-transcriptional level and influence many processes in insects, including metamorphosis, development, and insecticide resistance, thus offering a novel and sustainable pest management strategy.

RNA-based strategies are being harnessed to generate amiRNAs capable of silencing vital insect genes and preventing plant pests. Aphids are a pest that affects a large number of high-value crops. In this context, Guo et al. [135] created a transgenic tobacco plant via amiRNA-based technology to confer resistance against the aphid *Myzus persicae*. Specific genes of *M. persicae* were used to construct amiRNA- and hairpin RNA (hpRNA)- expressing vectors. Among them, the gene *MpAChE2* was highly relevant since it encodes the enzyme acetylcholinesterase. This enzyme is significant to the insect’s central nervous system as it catalyzes the breakdown of the neurotransmitter acetylcholine to clear the synaptic cleft. Impairment in acetylcholine leads to muscle atrophy, paralysis, and even the aphid’s death. Concurrently, the plant amiRNA expression cassettes were inserted into pCAMBIA1300 to generate the plasmids. Transgenic tobacco plants were then generated via *A. tumefaciens*-mediated transformation. Upon further growth, the plants were inoculated with three adult aphids, which were subsequently removed after 5 days upon observation of offspring production. Quantification of the presence of insects was then conducted, and a reduction in nymph production by up to 32% was noted in adult aphids. Although favorable outcomes were evident with the hpRNA approach, slightly better insect resistance was observed in transgenic plants employing RNAs derived from pre-amiRNA targeting the same *MpAChE2* gene [135]. In summary, this report suggests that amiRNA-based strategies are functional to reduce the effects of pests in plants, and amiRNAs may exhibit better performance than other RNA-derived strategies.

In a similar study conducted to enhance resistance in tomato plants against *M. persicae*, Faisal et al. [136] constructed an amiRNA vector targeting *M. persicae acetylcholinesterase* 1 gene (*Ace1)*, which also encodes the acetylcholinesterase enzyme. *A. tumefaciens* was used for the genetic transformation of tomato plants, which effectively integrated and expressed the amiRNA. The results showed that *Ace1* gene expression levels were reduced in aphids fed on transgenic plants compared to those of the control, and a significant decrease in the aphid population was also noticed.

The *Helicoverpa armigera* species of moth can infest more than 30 agricultural crop species. Agrawal et al. [137] used amiRNA technology against the chitinase gene of the pest, as this gene is critical for the molting of the insect. amiR-24 was developed to target the chitinase gene, and it was introduced into the pCAMBIA2300 vector with the CaMV 35S promoter. A tobacco plant was used as a vector host to study the effect of amiR-24 on *H. armigera* after consumption of its leaves. For 5 days, 10 neonatal larvae of *H. armigera* were provided with leaves from 2-month-old transgenic tobacco plants. The results indicated that control plants suffered significantly more damage than those expressing amiR-24. Similarly, surviving larvae fed with transgenic leaves showed lower weight than initially recorded, and mortality rates of up to 6 out of 10 were achieved after 5 days of infestation. Moreover, the amount of chitin expressed by larvae fed with control leaves versus those fed with transgenic leaves expressing amiR-24 was analyzed. Chitin expression was downregulated in larvae fed with the leaves expressing amiR-24 compared with the control. Subsequently, larvae with low chitin levels ceased molting, whereas control larvae exhibited normal growth and regular processes. In addition, transgenic tobacco plants exhibited growth and adaptation without any abnormalities. Over time, insects have evolved and overcome current insecticidal or pest control strategies and amiR-24 represents a novel alternative that targets specific insect biological functions without damaging crops [137].

Saini et al. [138] conducted a study to create a novel strategy to manage *H. armigera*, a pest that causes significant damage to crops such as chickpeas, soybeans, and pigeon peas, leading to billions of dollars in losses annually. The research involved silencing the *HaAce1* gene, which encodes the predominant isoform of acetylcholinesterase in *H. armigera*. This enzyme is essential for the correct transmission of nerve impulses, which coordinates various biological activities within the organism. Its absence leads to an interruption in the normal physiological activities of the organism. The proposed amiRNAs were carefully selected and screened to ensure that they would not inadvertently affect humans, plants, or beneficial insects. The selected amiRNA (HaAce1-amiR1) was ligated to the plant binary vector pBI121 and delivered into tobacco leaf disks by *Agrobacterium*-mediated transformation. Adventitious shoots from these explants were grown under the required conditions and eventually transferred for rooting. *H. armigera* were reared on Petri plates and fed on mature leaves of the grown transgenic tobacco lines. The insecticidal effect resulted in 25% mortality of the larvae. Approximately 20% of the larvae that reached the adult stage showed pattern deformities such as defective wings.

Insects of the order Hemiptera cause damage to various economically important crops, and the decline in the efficacy of current pest control methods, such as *Bacillus thuringiensis* (Bt) toxin, is a cause for concern. Bally et al. [139] chose the acetylcholinesterase gene 2 (*ACE2*) of *H. armigera* as a target because its inhibition reduces larval growth and causes malformation and mortality of the insect. amiRNA designs were based on five backbones from different insect species, i.e., Dm-ba (*Drosophila melanogaster*), Tc-ba (*Tribolium castaneum*), Dv-miR1, Dv-miR279 (*Diabrotica virgifera*), and At-miR159 (*Arabidopsis thaliana*). Four of these backbones were selected because they are miRNAs widely present in insects. This was based on the hypothesis that highly abundant miRNAs are efficiently processed by Drosha and Dicer-1, which play an important role in the maturation of miRNAs. The pre-miRNAs were replaced with an amiRNA sequence targeting the *H. armigera ACE2*. Accordingly, *Agrobacterium*-mediated transformation was executed to generate independent transgenic lines of *N*. *benthamiana. H. armigera* larvae were fed continuously with transformed *N. benthamiana* seedlings for 21 days. The results showed that most of the larvae fed on the transgenic plant failed to pupate and died, and the survivors took longer to pupate than those fed on WT plants. In addition, larvae fed on Dv-miR1 seedlings had a 60% mortality rate, and moths that emerged from the pupae of larvae that had fed on amiRNA plants showed significant developmental defects, particularly those whose larvae had ingested the Tc-ba-derived amiRNA [139]. The results of this study showed that the use of strategies that take advantage of the specificity of amiRNA-focused tools is effective in finding new ways to address problems that can affect the agricultural sector.

*H*. *armigera* also affects tomato plants. Consequently, Yogindran and Rajam [140] developed an amiRNA-centered strategy to generate tomato plants resistant to this pest by silencing the ecdysone receptor (*HaEcR*) gene of *H. armigera*, which plays an important role in the developmental stages regulation of the insect’s life cycle. *A*. *thaliana* miRNA precursor miR319a was used to generate the precursor backbone for the expression of amiRNA-*HaEcR*. The amiRNA-*HaEcR* sequence perfectly matched the one of the target *HaEcR* mRNA. Tomato plants were transformed with amiRNA-*HaEcR* via *A*. *tumefaciens*. Later, *H. armigera* larvae were fed for 10 days on detached leaves of the transgenic tomato plants, which stably expressed amiRNA-*HaEcR* after 72 h, and the other group was fed with untransformed control plants. After 5 days of continuous feeding, the mortality of larvae had a maximum level of 35% due to one line of the transgenic plants and 16–21% because of others, and there was no mortality in the ones fed with controls. After 8 days, 31–75% mortality was recorded due to transgenic tomato leaf feeding and around 23% as a result of control feeding, while 10 days of continuous feeding of transgenic tomato leaves led to approximately 92% mortality caused by two transgenic lines and 70–83% by the others. Control feeding displayed around 43% mortality [140].

The soybean cyst nematode (SCN), *Heterodera glycines*, is one of the most destructive parasites of the soybean plant. To combat this pest, Tian et al. [141] investigated the effectiveness of using pest-derived amiRNA methods. For this study, three genes that are related to the reproduction of these nematode parasites were selected. One gene was *J15*, which is involved in embryogenesis and egg production, while the *J20* gene is related to a phosphatase precursor that influences a variety of cellular responses and signaling processes. Moreover, the *J23* gene is essential for the cytoskeletal structure of the SCN. Accordingly, three amiRNA sequences were developed: pUFamiR-J15, pUFamiR-J20, and pUFamiR-J23. Hairy roots of soybean plants were transformed and grown for bioassays for up to 3–4 weeks. Transgenic plantlets were transplanted into contaminated soil containing SCN eggs. The total number of SCN cysts and eggs remaining on each plant was counted after five weeks of growth. The highest reductions in cyst numbers per gram of root were 28% for the amiR-J15 lines and 43% for the amiR-J23 lines, in comparison to control plants. Egg densities followed a similar pattern, with the amiR-J15 and amiR-J23 lines showing reductions of 53% and 47% in this matter, respectively. In summary, this strategy demonstrated the ability to interfere with the integrity and reproductive capacity of SCN cysts and eggs, providing an attractive mechanism against pathogens. However, a potential limitation could be the variability in SCN survival rates due to mixed transgenic hair roots [141].

The whitefly (*Bemicia tabaci*) is an important insect pest that affects various economically important crops. Besides feeding on plants, sucking their sap, and damaging and depriving them of vital nutrients, this insect transmits plant viruses. Zubair et al. [142] developed a plant amiRNA expression approach against whiteflies by targeting three of their essential genes, namely *Sex-letha*l (*Sxl*), *Acetylcholinesterase* (*AChE*), and *Orcokinin* (*Orc*). *Sxl* is important for the sex determination cascade of the insects; *AChE* is a neurotransmitter whose function blockage leads to muscular dysfunction, paralysis, and even insect death; and *Orc* regulates the circadian clock that controls movement activity in the insects. The *A*. *thaliana* miR159 backbone precursor was engineered to express the amiRNAs and *N*. *tabacum* plants were transformed with the construct via *Agrobacterium*-mediated transformation. Four weeks after whitefly infestation of transgenic and control tobacco plants, it was observed that the number of whiteflies was significantly reduced on transgenic plants and had notably increased on control plants. The control plants displayed thickening, leaf curling, and stunted growth symptoms, and the whiteflies fed on them developed normally. In contrast, there was a considerable decline in the number of eggs and whiteflies in the transgenic plants. Additionally, the expression of the target genes was significantly downregulated in whiteflies feeding on the transgenic *N. tabacum* plants compared to the ones feeding on the controls [142]. These observations indicated that the amiRNA approach successfully conferred resistance against whitefly by silencing the target genes *Sxl, AChE*, and *Orc*, thus being a promising strategy for generating whitefly-resistant crops.

The authors suggested that the larval mortality observed from feeding on control leaves may have resulted from factors such as metabolic abnormalities within the larvae population and the characteristics of the food consumed by the insects. In addition, larvae fed continuously with transgenic tomato plants displayed an important weight reduction (16–36%) after 8 days, as well as development delay. Feeding with transgenic tomato leaves also led to a reduction in HaEcR gene expression levels. Furthermore, all of the ecdysone signaling pathway genes presented a reduction in their expression levels, meaning that amiRNA-*HaEcR* also affected the expression of downstream genes. With these results, it was determined that higher mortality of larvae, weight reduction, and development delay were caused by amiRNA ingestion, leading to increased insect resistance in transgenic tomato plants compared to control plants. Finally, it was found that the transgene of transgenic tomato plants could be adequately inherited and expressed in their offspring [140]. In sum, this plant-mediated gene silencing approach through amiRNAs effectively targeted the gene of interest in *H. armigera* to enhance resistance against insect pests.

The use of amiRNAs has also progressed significantly in managing insect pests, providing a promising alternative for crop protection given the increasing resistance of pests to traditional methods such as pesticides and Bt toxins. Nonetheless, this application faces the challenge of scaling amiRNA-carrying plants to large agricultural fields. Additionally, crops are often attacked by multiple pest species, making it essential to explore multiplex amiRNA strategies that can target and control diverse pests simultaneously.

## 6. Applications of amiRNAs in Plant Metabolism

In plants, a number of miRNAs control the production of metabolites that affect growth, development, nutrient levels, and plant traits. Accordingly, the regulation of metabolic pathways can be controlled by amiRNA technology, using positive or negative feedback mechanisms. This may allow the inhibition of unwanted metabolic products and the enhancement of desired metabolites, as well as the regulation of metabolic signaling. Recently, the significant role of amiRNAs in regulating biosynthetic activities and metabolites in plants has garnered attention, especially in key crop species. Consistently, this approach could offer an effective strategy for improving plant development, fruit and seed production, and secondary metabolite yield, as well as enhancing properties in non-food or biofuel crops.

Polyphenol oxidase (PPO)-catalyzed oxidation is responsible for 50% of the total losses in the industrial production of fruits and vegetables. PPO in *Solanum tuberosum L*. is encoded by a multigene family. One of the advantages of amiRNA technology is its capability to silence individual genes or partially silence a multigene family with low off-target adverse effects. Chi et al. [143] used different amiRNAs on *S. tuberosum* to functionally characterize members of the PPO gene family, specifically *StuPPO1*, *StuPPO2*, *StuPPO3*, and *StuPPO4*, individually or in combination. In total, seven amiRNAs were developed and labeled according to the gene targeting: amiRPPO1, amiRPPO2, amiRPPPO3, amiRPPO23, amiRPPO234, amiRPPO234A, and amiRPPO1234. By integrating these amiRNAs individually into the potato genome using a plant binary vector, transgenic potato lines were successfully generated. PPO gene expression, PPO protein levels, PPO enzymatic activity, and browning potential were measured. These lines showed significant downregulation of the targeted PPO genes, with reductions in transcript expression levels ranging from 68% to 98%. Simultaneous silencing of *StuPPO1*, *StuPPO2*, *StuPPO3*, and *StuPPO4* genes in three amiRPPO1234 transgenic lines resulted in a 70–80% reduction in tuber PPO protein levels. Consequently, PPO enzymatic activity was decreased by approximately 90%, and the potential for browning was reduced by 70–75%. The suppression of PPO genes significantly decreased PPO activity and enzymatic browning in potatoes, addressing a key issue that impacts their commercial and nutritional value [143]. These findings demonstrate the potential of amiRNA technology to enhance crop quality by precisely controlling the expression of multiple genes. Hence, this approach offers a promising tool for future advancements in plant metabolic engineering and agriculture.

*Phaeodactylum tricornutum* is a model organism for pennate diatoms in which few miRNAs have been detected. Kaur and Spillane [144] aimed to demonstrate the efficacy of amiRNAs in reducing the expression of the phytoene synthase gene (*PSY*), which plays a critical role in carotenoid biosynthesis, thereby addressing the problem of manipulating metabolic pathways in diatoms. The amiRNA foldback cassette was assembled from four oligonucleotide sequences under the fucoxanthin chlorophyll a/c-binding protein A promoter (FcpA) to obtain the final amiRNA expression vector pPhaT1-GFP-amiRNA-PSY. Four transgenic *P. tricornutum* independent lines were studied: amiRPtT1, amiRPtT2, amiRPtT5, and amiRPtT9. Lines carrying amiRNAs showed higher levels of amiRNA expression and a significant reduction in PSY mRNA expression levels compared to WT plants. In particular, amiR-PTt9 exhibited the highest transgene copy number and was correlated with the highest levels of amiRNA expression amongst the four transformants. The results confirmed that the diatom’s RNA silencing machinery can efficiently process amiRNAs effectively, leading to targeted gene silencing. Moreover, the levels of carotenoids in the transformed lines were decreased when compared to WT specimens. This research highlights the versatility and effectiveness of amiRNAs in modifying metabolic pathways, setting the stage for future advances in plant and algal biotechnology.

Shafrin et al. [145] worked on *Corchorus olitorius* (jute) to reduce the lignin content in its fibers and improve its economic and environmental viability. Lignin is a major obstacle to the use of jute for various applications due to its negative impact on fiber quality, making the fiber less suitable for various industrial applications, including textiles and bioplastics. amiRNAs were used to specifically target and silence the coumarate 3-hydroxylase (*C3H*) and ferulate 5-hydroxylase (*F5H*) genes, key players in the monolignols pathway (a series of biochemical steps that produce monolignols, the building blocks of lignin). The methodology of the study involved the design of amiRNA constructs (C3H-amiRNA and F5H-amiRNA) followed by *Agrobacterium*-mediated transformation of jute plants. The amiRNA transgenic lines showed an approximately 25% reduction in acid-insoluble lignin content in the whole stem and a 13% reduction in fiber lignin content compared to non-transgenic plants. This study demonstrates a viable pathway for increasing the commercial value of jute and reinforces the application of amiRNAs to specific metabolic challenges in plants.

The green alga *C*. *reinhardtii* is a promising source of H_2_ for a new generation of renewable energy. Li et al. [146] applied amiRNA-based technology to target the oxygen-evolving enhancer 2 (*OEE2*) gene, a photosystem II-related protein, to overcome the challenge of simultaneous O_2_ and H_2_ evolution, which hinders efficient H_2_ production. A heat-inducible expression vector containing amiRNA-OEE2 was constructed and transformed into *C. reinhardtii*. Upon induction, the transgenic algae showed a significant increase in O_2_ consumption, decreasing its concentration from 22% to 16%. Meanwhile, the H_2_ production yield increased to approximately twice that of the control group following a second heat shock, indicating a substantial improvement in the production of this valuable source of energy. Later, in a similar study, Li et al. [147] designed a heat-inducible amiRNA expression system targeting the transcript of the D1-encoding gene (*psbA*). The D1 protein is a member of the photosystem II (PS II) complex of *C. reinhardtii*. The inhibition of this gene has previously been associated with improved H_2_ yield and suppression of photosynthetic O_2_ evolution. After a 7-day culture period following heat induction treatment, transgenic lines expressing amiRNA-D1 showed a 60% increase in H_2_ production compared to the control group. The results of both studies demonstrate the potential of amiRNA technology to modulate gene expression in inducible systems and to avoid irreversible effects that persist in cells. These alternatives may provide sustainable and advantageous methods for optimizing the industrial production of H_2_.

In contrast, Wang et al. [148] proposed a different method for H_2_ production on *C. reinhardtii* than the heat shock-inducible system, which might have fewer undesired effects on cell growth and is less difficult and cost-effective to control in large-scale cultures. A blue light-inducible gene expression system was used to induce transcription levels of an amiRNA (amiR-D1). Blue light allows heterodimerizing proteins (CIB1 and CRY2) to interact and bring GAL4 BD and VP16 together. A specific DNA upstream activation sequence (UAS) is bound by GAL4 BD and enables amiR-D1 gene transcription. The amiR-D1 target gene is the PS II reaction center protein D1 (*psbA*), a key part of hydrogen production. *C. reinhardtii* strain was transformed using the glassbead vortexing method with two vectors: pDb124-Gal4 BD-CIB1 and pDh124-VP16-CRY2-UAS-amiRNA. Transgenic algae treated with blue light produced twice the amount of hydrogen compared to the control group and showed no difference when treated with white light. However, it was found that only the initial light irradiation resulted in a significant increase in hydrogen yield. The lack of available nutrients in the medium after the cell growth and division stage may not have been sufficient to enable cells in a poor growth state to generate enough electrons for the photosynthetic electron transfer chain in hydrogen production. This work is highly relevant to the advancement of sustainable bioenergy solutions. In addition, it presents a light-inducible exogenous gene expression system using amiRNA technology, which can be highly versatile because all functional components of the system, including DNA binders and light receptors, can be swapped for different targets, for example, to activate or repress transcription.

Besides being a remarkable source of H_2_, *C. reinhardtii* has the potential for the production of biodiesel due to its favorable characteristics, such as high growth rate and high oil production. However, it is crucial to increase the lipid content for optimal commercial-scale production in the future. Nitrogen depletion has been a common way to stimulate fatty acid production in this species, but it might produce undesirable effects on algal growth and even cell death. Consequently, Wang et al. [149] attained the inhibition of phosphoenolpyruvate carboxylase (PEPC) in *Chlamydomonas* using amiRNAs. PEPC catalyzes the formation of oxaloacetate from phosphoenolpyruvate and regulates carbon flux. In this way, carbon can be directed to fatty acid synthesis rather than to other metabolic pathways. Two subtypes of CrPEPC enzymes, CrPEPC1 and CrPEPC2, were targeted by amiRNA-PEPC1 and amiRNA-PEPC2, respectively. Both were introduced into *C. reinhardtii* strains by a glass bead method and subjected to heat shock (HS) treatment. Total fatty acids (TFA) were extracted from transgenic mutants and WT, showing an increase in almost all the different fatty acids. The expression of amiRNAs in transgenic *C. reinhardtii* was increased approximately 28-fold after HS, and an increase in TFA of 28.7–48.6% was detected. Accordingly, amiRNA technology has enabled the manipulation of fatty acid synthesis in *C. reinhardtii*, which can be helpful to successfully meet the demands of industrial biodiesel production and may help overcome current and future difficulties in wider applications.

An amiRNA (amiFATB) was designed by Ozseyhan et al. [150] to downregulate genes encoding the fatty acyl-ACP thioesterases (FATB) in *Camelina sativa*, a promising oilseed crop that is being actively studied primarily for bioenergy generation but with low-quality oil due to significant levels of undesired long-chain, saturated, and polyunsaturated fatty acids. In this study, the mature miRNA sequence of Csa-miR159a was replaced within the precursor backbone with a gene-specific sequence tailored to target the FATB encoding gene, a crucial factor in the buildup of saturated fatty acids in oilseeds; this amiRNA was named amiFATB. As a result, significant downregulation of *FATB* gene transcripts in developing seeds was observed, coupled with about 45% of low palmitate (16:0) and 38% of stearic acid (18:0) reduction. In addition, the total saturated fatty acid content decreased by 35%. The knockdown of *FATB* transcripts in a high-oleic line with the amiRNA resulted in a decrease in saturated fatty acids and increased accumulation of oleic acid; its content went from roughly 62% to over 70%, thus providing an added biotechnological tool for the purpose of improving seed oils for chosen properties and the improvement of oil qualities.

As noticed, the use of amiRNAs in plant metabolic engineering is still in its infancy, primarily focused on enhancing H_2_ production in specific plant species. Notwithstanding this, given the advancements in the development of amiRNA backbones for various plant species, amiRNA technology should be expanded to boost the production of secondary metabolites with medicinal properties in economically important plants rather than being limited to biofuel production.

## 7. Applications of amiRNAs Against Drought Stress

amiRNA-based technology also offers a precise method for enhancing plant resilience to drought and other stresses via targeting specific genes involved in stress-related signaling pathways. Particularly for drought stress, amiRNAs can be tailored to downregulate genes that negatively modulate water retention, root development, or abscisic acid signaling, directly bolstering the plant’s water-use efficiency and drought tolerance. Nevertheless, just a couple of studies have been conducted in this matter up to date.

In 2016, Wyrzykowska et al. [151] developed a protocol for preventing drought stress in *S*. *tuberosum* by targeting the *CBP80/ABH1* gene. Such a gene is a component of the Cap Binding Complex (CBC), which participates in mechanisms of response to drought stress in plants. The corresponding amiRNAs were designed in the WMD2 platform and were called amiRNA80.1 and amiRNA80.2. Subsequently, transgenic potato plants were generated via *Agrobacterium*-mediated transformation. Remarkably, transgenic plants displayed decreased expression levels of *CBP80/ABH1* and improved tolerance to water shortage. Therefore, these observations indicate the prospective use of amiRNA-mediated approaches to manage drought stress in important crops.

**Figure 2:**
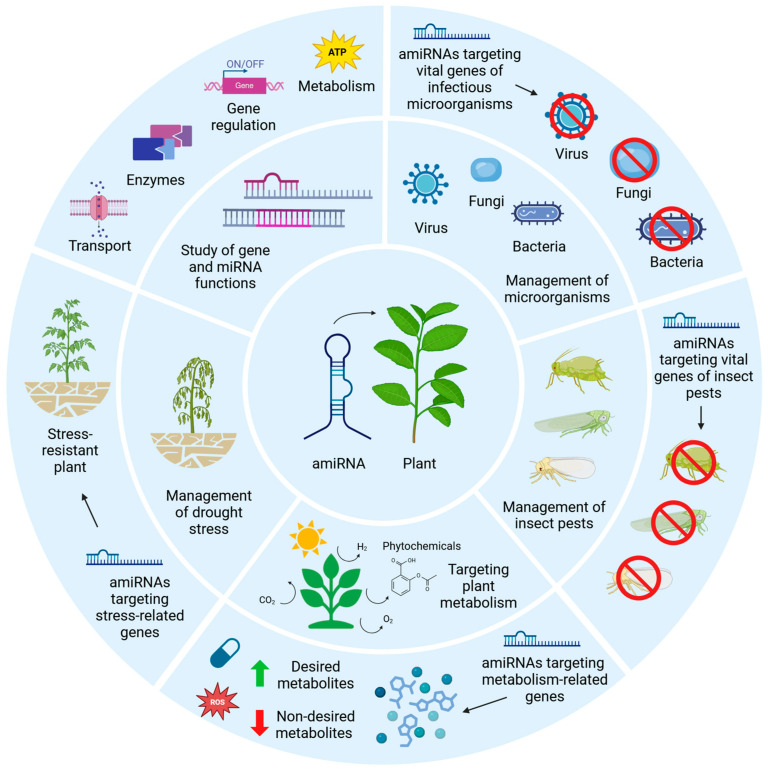
Current applications of amiRNA-based approaches in plant biotechnology. amiRNAs are utilized to study the functions of genes and miRNAs, offering insights into complex regulatory networks. In addition, amiRNAs can be employed to combat infectious diseases caused by viruses, fungi, and bacteria, enhancing plant health. They also play a significant role in pest management, providing an alternative to traditional methods by targeting insect pests with precision. In metabolic engineering, amiRNAs contribute to the optimization of plant metabolism, boosting the production of valuable secondary metabolites while reducing the synthesis of undesirable compounds. Furthermore, amiRNAs can help to produce plants that can withstand abiotic stress conditions, such as drought, thus supporting agricultural productivity under challenging environmental conditions (created with a licensed version of Biorender.com).

In a similar study centered on inducing drought tolerance in potatoes, the proline dehydrogenase (ProDH) enzyme was identified to be implicated in drought stress response. Particularly, the expression of two of the genes responsible for encoding this enzyme (*StProDH1* and *StProDH2*) was tissue-specific and varied under stress conditions, including water shortage. Importantly, ProDH is crucial in proline catabolism and since proline is involved in the mechanisms of drought stress response, the inhibition of its catabolism may contribute to enhanced resistance to water deprivation. To explore this further, an amiRNA targeting *StProDH1* (the gene that was detected to participate in most of the stress conditions) was designed, driven by the CaMV 35S promoter, and introduced into potatoes via *Agrobacterium*-mediated transformation. As a result, silencing *StProDH1* led to increased proline in transgenic plants under drought, helping them maintain better physiological parameters like malondialdehyde, water, and chlorophyll content compared to non-transgenic plants. Further, after rehydration, transgenic plants retained higher proline and sugar levels, underscoring the role of *StProDH1* in drought tolerance in potatoes [152]. A summary of the applications of amiRNAs in plant biotechnology is depicted in Figure 2.

## 8. Concluding Remarks

The intricate challenges posed by our evolving global landscape in ensuring food security and preserving plant diversity have undeniably sped up the development of novel technologies geared toward tailored plant breeding. In this regard, naturally occurring miRNA-mediated regulatory mechanisms of gene expression in plants have inspired the conception of innovative technologies based on amiRNAs, the artificial counterparts of miRNAs, which can be tailored to hinder the expression of transcripts of interest with great accuracy. Thus, over the last few decades, amiRNA-based regulation of gene expression has emerged as a prospective strategy to understand the functions of those plant miRNAs and genes whose biological implications are still ambiguous, improve pathogen resistance, enhance stress adaptability, and engineer plant metabolism to heighten the yield of valuable phytochemicals. Notwithstanding the above, further research is needed to fully optimize the effectiveness of amiRNAs through a comprehensive understanding of the endogenous mechanisms governing the biogenesis and processing of plant miRNAs. Furthermore, there is a need to delve into the principles and requisites necessary for the accurate occurrence of miRNA-guided gene modulation, as these features are crucial to ensure the functionality of amiRNAs, particularly in non-model plants. Thus, further advancements in the pivotal field of amiRNA-based technology could pave the way for enhancing our knowledge of the fundamentals of plant miRNA biology, facilitating the assisted development of elite cultivars.

## 9. Future Prospects

As evidenced throughout this review, the emergence of amiRNA technology in recent decades has initiated a transformation in the field of plant biotechnology. However, despite their potential, further research and development are needed before amiRNA-based approaches can be widely adopted for plant breeding. Moreover, it is important to approach this technology with cautious optimism, recognizing that there are several challenges yet to be addressed and improvements to be made.

For instance, the adoption of amiRNA technology by farmers faces significant challenges. The production of transgenic plants harboring specific amiRNAs may be restricted by high costs, which include the generation, testing, and commercialization of genetically modified organisms (GMOs). These costs are particularly prohibitive for small-scale farmers or those in developing regions, where financial constraints may limit access to this technology [153,154,155]. Furthermore, the legal and regulatory frameworks governing the use of GMOs vary widely across countries, with some imposing strict limitations or outright bans [156,157]. Consequently, such regulations can slow down the approval process and increase costs, further impeding the implementation of plant amiRNA technology. A possible solution for this lies in the generation of methodologies centered on the transient expression of amiRNAs through topical administration, avoiding the need for stable genetic modifications that can raise ethical and regulatory concerns. Techniques such as those employed for topically delivering double-stranded RNAs (dsRNAs) [158,159] and siRNAs [160], which involve spraying or rubbing leaves with an RNA-containing solution, have shown promise in delivering RNA-based treatments directly to plants. Additionally, the feasibility of topically applying plasmids capable of expressing amiRNAs to achieve transient expression has already been evidenced in *N*. *benthamiana* and *S*. *lycopersicum* [68].

However, since current experiments studying the potential of plant amiRNAs have only been carried out at the laboratory level, there is great uncertainty about the risks associated with the widespread use of this technology in crop fields and its impact on the environment. One major concern is the potential for resistance development in pests and pathogens. Indeed, prolonged exposure to amiRNA-mediated silencing could exert selective pressure, leading to the evolution of resistant strains that either bypass the target pathway or mitigate the effect of the silencing mechanism, as previously stated for other RNAi-based approaches [161,162,163,164]. Accordingly, the potential emergence of resistance against amiRNA-based pest/pathogen control strategies could undermine the long-term efficacy of amiRNA-based strategies and would require the development of tailored tactics to minimize resistance, such as the use of multiplex amiRNAs targeting various genes or pathways simultaneously, as has also been suggested for RNAi-centered technologies [165].

Another critical issue is the risk of unintended consequences, including off-target effects that may affect non-target organisms, such as beneficial insects, soil microbiota, or neighboring plant species [157,166,167,168]. Additionally, concerns about the potential horizontal gene transfer (HGT) of amiRNA sequences to non-target organisms raise ecological and biosafety questions. HGT could inadvertently spread the silencing capability beyond the intended scope, potentially disrupting local ecosystems [169]. To mitigate these risks, rigorous ecological assessments, precise amiRNA design to ensure high specificity, and the exploration of transgene-free delivery systems should be prioritized to enhance the safety and acceptance of amiRNA technologies in agriculture. Nonetheless, further research is required to comprehend the elusive effects of plant amiRNAs on ecosystems. Hence, such understanding can only be achieved by analyzing the environmental impact of amiRNAs in isolated agricultural fields that can mimic real-world conditions and provide a controlled environment for experimentation without posing risks to surrounding ecosystems.

As aforesaid, the use of the transient, transgene-free expression of amiRNAs in plants holds significant importance in plant biotechnology. This approach can facilitate the targeted and temporary modulation of gene expression, offering a versatile tool for studying gene and miRNA functions and improving plant traits without the long-term presence of exogenous genetic material [70,83]. One key advantage of transient expression is its potential to avoid regulatory hurdles associated with the production of transgenic plants, which simplifies regulatory approval processes and accelerates research timelines [170,171]. In this regard, Lunardon et al. [67] transiently introduced polycistronic amiRNAs in *S*. *lycopersicum* and *N. benthamiana*, while Cisneros et al. [82] employed a transgene-free, virus-based approach to express amiRNAs originating from minimal precursors in *A. thaliana* and *N. benthamiana*. In addition, a relevant investigation performed by Zhang et al. [69] demonstrated that amiRNAs could also be delivered to plant cells without the requirement of viral- or bacterial-based approaches via Au nanoparticles to effectively silence target genes in *A. thaliana*. Hence, these reports shed light on the fact that transgenic-free expression of amiRNAs could also become a widely adopted technique in the future. Nonetheless, the use of inducible promoters for amiRNA expression should also be explored in more detail in the future. An example of this strategy was reported by Flores-Sandoval et al. [172] due to the fact that the constitutive expression of the amiRNAs designed by them provoked sporeling lethality in *Marchantia polymorpha*. Therefore, to avoid this undesired effect, they used a system based on the estrogen-responsive chimeric XVE transcription factor, whose functions are induced by the presence of β-estradiol.

It is worth mentioning that the use of gene insertion (also known as gene knock-in) through the genome editing tool CRISPR/Cas has not yet been exploited in this research arena. Mechanistically, the CRISPR/Cas system can mediate directed double-strand DNA breaks (DSBs) in a targeted genomic region, triggering DNA repair pathways, i.e., non-homologous end joining (NHEJ) or homologous DNA repair (HDR). Therefore, in the event that a template containing a transgene (in this case, the expression cassette of the amiRNA) is in close proximity during the repair process, there exists a likelihood that this transgene may be integrated into a DSB, resulting in the insertion of a new gene into the designated genomic site [173,174,175]. As a matter of fact, the CRISPR/Cas9 knock-in approach has already been applied in diverse plant species, such as *C*. *reinhardtii* [176], *N*. *tabacum* [177], rice [178], and petunia [179]. Even so, to date, targeted knock-in or gene replacement strategies have not been utilized in CRISPR/Cas-mediated studies of plant ncRNAs [180]. To achieve the successful insertion of amiRNAs (indeed, any gene in general) into the genome using CRISPR/Cas, specialized protocols and designs are essential. These protocols should enable the targeted delivery of the gene to transcriptionally active regions of the genome, the generation of suitable donor templates, and the precise temporal and spatial coordination between the occurrence of the DSB and the supply of the donor template [173,181,182].

Although CRISPR/Cas technology coupled to miRNA-based approaches has been limited to editing endogenous miRNA genes in plants to date [180], noteworthy reports in mammalian cells on the use of endogenous miRNAs or exogenous amiRNAs for the optimization of CRISPR/Cas indicate that these types of strategies could be extrapolated to plant systems in the future. As an example, Hirosawa et al. [183] generated a miRNA-responsive AcrllA4 switch that allows the modulation of CRISPR/Cas activity in a cell-specific manner by adding a miRNA target site to the 5′UTR of AcrIIA4 mRNA. The target site included in the mRNA corresponded to that of a miRNA highly expressed in the treated cells (i.e., miR-21-5p for HeLa cells). Given that AcrIIA4 is an anti-CRISPR protein that blocks the binding of Cas9 to DNA, when miRNA activity is low, AcrIIA4 is expressed, thus blocking the genome editing functions of the CRISPR/Cas9 complex. In contrast, high miRNA activity suppresses AcrIIA4, enabling Cas9 to bind DNA and edit the target gene in specific cells. Likewise, a cell type-specific switch was generated by Hoffmann et al. [184] by incorporating target sites for miR-122 and miR-1 into the 3′UTR of AcrIIA4 mRNAs to enable selective knockdown of the anti-CRISPR protein in liver and cardiac muscle cells, respectively. This approach allowed Cas9 activation exclusively in hepatocytes or cardiomyocytes, while maintaining effective Cas9 inhibition in non-target cells. Considering that the efficacy of the anti-CRISPR AcrIIA4 protein has already been evidenced in the model plant *N. benthamiana* [185], as well as in *A. thaliana* and in the hybrid poplar “717” [186], amiRNA technology could enable precise regulation of CRISPR/Cas-mediated genome editing in plants through the design of cell-specific amiRNAs and corresponding amiRNA recognition sites within anti-CRISPR protein mRNAs.

In an interesting miRNA-responsive CRISPR/Cas mechanism proposed by Yun and Jung [187], a DNA regulator binds to the ribonucleoprotein (RNP) complex, forming an RNP–regulator complex that remains inactive even in the presence of target DNA. The liberation of the RNP complex happens only in the presence of a specific miRNA that binds to a toehold sequence in the DNA regulator through complementary base pairing. This interaction displaces the DNA regulator from the RNP complex, reactivating the RNP and enabling it to recognize and cleave the target DNA. Apart from that, Huang et al. [188] discovered that amiRNAs can control the CRISPR/Cas system by targeting single-guide RNAs (sgRNAs) in HEK-293T cells and C57Bl/6 mice. While amiRNAs did not directly reduce sgRNA expression, they effectively disrupted sgRNA/Cas9 binding and inhibited Cas9 activity. This regulation was enhanced by the drug enoxacin, allowing precise and reversible control of CRISPR activity through adjustable enoxacin concentrations. Under these premises, researchers should explore the integration of amiRNA and CRISPR/Cas systems as a promising strategy for future applications in plant genetic engineering.

Intriguingly, over the past years, compelling evidence has indicated the existence of plant pri-miRNAs containing functional small ORFs that encode small peptides known as miRNA-encoded peptides (miPEPs). Similarly to miRNAs, miPEPs have important regulatory roles implicated in stress responses, growth, and development [189,190,191]. Nevertheless, several regulatory functions of miPEPs, as well as their corresponding mechanisms of action, remain elusive [192]. Thus, the potential use of amiRNAs to study miPEPs’ functions and design artificial miPEPs represents a promising avenue in plant biotechnology. For example, pri-amiRNAs can be crafted and delivered into plants to upregulate the expression of endogenous known miPEPs and gain valuable insights into the roles of these peptides in plant development, stress responses, and other physiological processes [193,194], as well as understand their biosynthesis and processing mechanisms. Furthermore, amiRNAs can be engineered in forthcoming investigations to encode artificially crafted miPEPs with tailored functionalities that might be beneficial for advancing our understanding of plant biology and developing innovative strategies for crop enhancement.

Additionally, amiRNA-mediated approaches could be harnessed to improve the nutritional content and phytochemical production of plants for pharmaceutical purposes. By targeting specific genes involved in the biosynthesis of key nutrients or bioactive compounds, such as vitamins, antioxidants, and flavonoids, amiRNAs can potentially modulate their levels in plant tissues [51,54,195,196]. Indeed, amiRNAs could be exploited to modulate the levels of essential nutrients in crops, addressing malnutrition or dietary deficiencies in human populations, as demonstrated in an investigation in which amiRNAs were designed to silence the expression of the *myo*-inositol kinase gene (*OsMIK*) in rice and reduce the production of phytic acid (an anti-nutrient that can reduce the bioavailability of micronutrients), thus generating low-phytic-acid rice [197]. Analogously, these techniques could be used to enhance the production of phytochemicals with pharmaceutical properties, such as anticancer or antimicrobial compounds, in plant species used for medicinal purposes [198,199].

Another noteworthy prospect that deserves the attention of molecular biologists relies on the application of amiRNA-centered strategies in the exploration of miRNA trafficking and functions within plant organelles that enclose their own genetic material, such as mitochondria and chloroplasts [200]. These organelles play crucial roles in energy production and metabolism [201], and it has been shown that certain classes of ncRNAs, including miRNAs, regulate the gene expression of these organelles [202,203]. On this basis, amiRNAs could be tailored to scrutinize miRNA biogenesis in the chloroplasts and mitochondria, unveil the functions of mitochondrial- and chloroplast-encoded miRNAs, and help understand miRNA import/export pathways between the cytoplasm and the abovementioned organelles; three major concerns that remain ambiguous in the panorama of plant ncRNAs [204,205,206]. As a result, these endeavors may lead to a deeper understanding of how miRNAs regulate gene expression within organelles and how these processes impact plant growth, development, metabolism, and stress responses. Nonetheless, challenges such as the delivery of amiRNAs to organelles and the potential off-target effects of these molecules will need to be addressed to fully exploit the potential of amiRNA-centered strategies in organelle research.

Importantly, the effectiveness of amiRNA technology is not limited to specific plant species but is instead influenced by the efficiency of genetic transformation and, most critically, by the design of the pre-amiRNA. Properly tailoring the pre-amiRNA to resemble the secondary structure of highly conserved endogenous miRNAs is essential to ensure its effectiveness in a particular plant species. While some plants are more recalcitrant to genetic transformation, this obstacle can be overcome using transient expression methods [207], which facilitate amiRNA-mediated gene silencing without the necessity of creating transgenic lines. Furthermore, as highlighted in previous sections, the adaptable design strategy of amiRNAs has been successfully applied across a variety of plant species, underscoring the potential for amiRNA technology to be implemented, to the best of our knowledge, in virtually any plant. Notwithstanding this, it is worth considering that although phylogenetically conserved pre-miRNA backbones are commonly used, it is generally preferable to utilize backbones derived from the plant species of interest [51].

**Figure 3:**
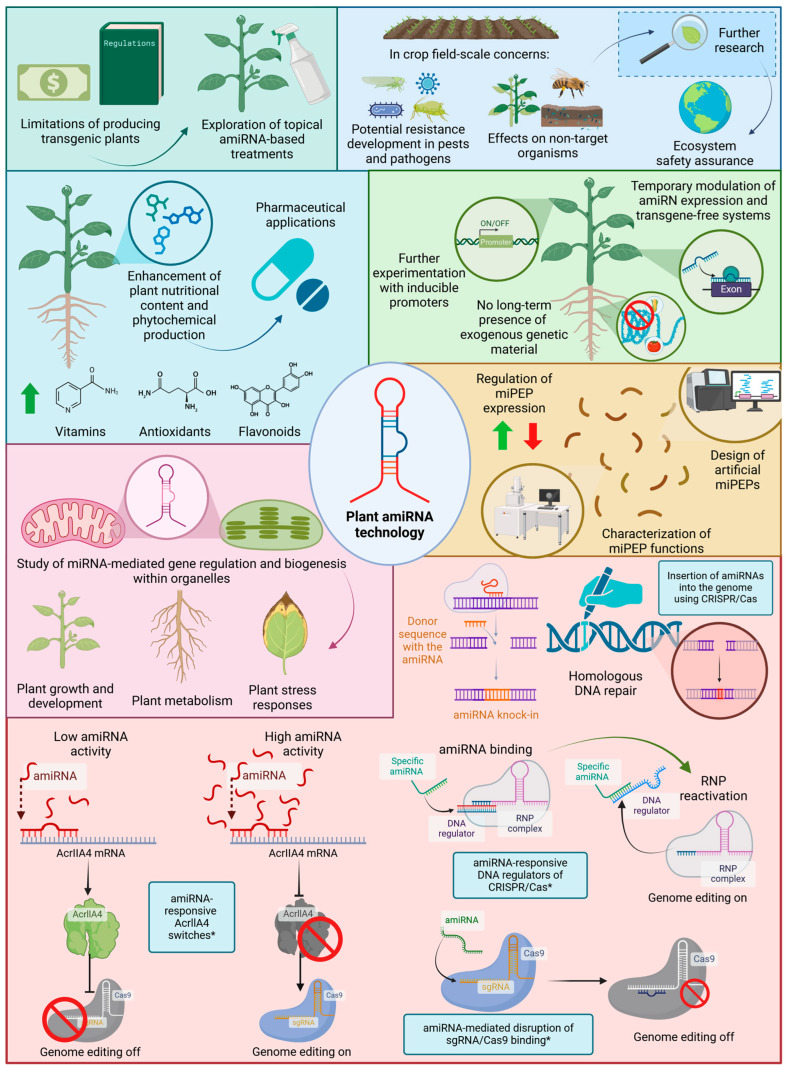
Future insights into amiRNA-based approaches applied in plant biotechnology. Scientists worldwide must seek solutions to overcome the economic and regulatory challenges associated with this technology, while also investigating its potential environmental impact and effects on non-target species. Upcoming investigations on amiRNAs should also focus on targeting plant metabolism to enhance the production of phytochemicals, exploring the temporal modulation of amiRNA expression, and developing transgene-free systems for amiRNA delivery. Studies should also investigate the functions of those miPEPs encoded by certain plant miRNAs, examine the roles and processing of organelle-localized miRNAs, and consider the potential integration of amiRNA and CRISPR/Cas technologies. * Proposals based on studies in mammalian systems. (created with a licensed version of Biorender.com).

Overall, we believe that the insights and remaining challenges discussed in this paper will be highly valuable in understanding the roles of plant miRNAs through amiRNA-based tools. Similarly, we anticipate that the use of amiRNA-mediated gene silencing might significantly expedite crop enhancement and the sustainable production of important plant-based compounds, which are critical for the pharmaceutical, agricultural, and food industries. However, the widespread adoption of transgene-free amiRNA technology is crucial for fully realizing amiRNA’s transformative potential in these fields. The key future perspectives of plant amiRNA-based technologies are illustrated in Figure 3.

## Data Availability

No new data were created or analyzed in this study. Data sharing is not applicable to this article.
